# Structure-Based Virtual Screening of Benzaldehyde Thiosemicarbazone Derivatives against DNA Gyrase B of *Mycobacterium tuberculosis*

**DOI:** 10.1155/2021/6140378

**Published:** 2021-12-13

**Authors:** Andiyappan Kistan, Balakrishnan Anna Benedict, Sundaramoorthy Vasanthan, Alphonse PremKumar, Malathi Kullappan, Jenifer Mallavarpu Ambrose, Vishnu Priya Veeraraghavan, Gayathri Rengasamy, Krishna Mohan Surapaneni

**Affiliations:** ^1^Department of Chemistry, Panimalar Institute of Technology, Poonamallee, Chennai 600 123, Tamil Nadu, India; ^2^Department of Research, Panimalar Medical College Hospital & Research Institute, Varadharajapuram, Poonamallee, Chennai 600 123, Tamil Nadu, India; ^3^Department of Biochemistry, Saveetha Dental College & Hospitals, Saveetha Institute of Medical and Technical Sciences, Saveetha University, Chennai 600 077, Tamil Nadu, India; ^4^Departments of Biochemistry, Molecular Virology, Research, Clinical Skills & Simulation, Panimalar Medical College Hospital & Research Institute, Varadharajapuram, Poonamallee, Chennai 600 123, Tamil Nadu, India

## Abstract

Emergence of antibiotic-resistant *Mycobacterium tuberculosis* (*M. tuberculosis*) restricts the availability of drugs for the treatment of tuberculosis, which leads to the increased morbidity and mortality of the disease worldwide. There are many intrinsic and extrinsic factors that have been reported for the resistance mechanism. To overcome such mechanisms, chemically synthesized benzaldehyde thiosemicarbazone derivatives were screened against *M. tuberculosis* to find potential inhibitor for tuberculosis. Such filtering process resulted in compound 13, compound 21, and compound 20 as the best binding energy compounds against DNA gyrase B, an important protein in the replication process. The ADMET prediction has shown the oral bioavailability of the novel compounds.

## 1. Introduction

Tuberculosis (TB) is a potentially serious communicable disease caused by the bacillus *Mycobacterium tuberculosis* (*M. tuberculosis*) [1]. TB was found with increased mortality, and it spreads from person to person through tiny droplets released into the air via coughs and sneezes [2]. According to the World Health Organization (WHO) report, worldwide, around 10 million people were affected with TB [3]. Here, 56% were men, 32% were women, and children accounted for 12%. Also, 1.2 million deaths were reported in HIV-negative people and 208000 deaths were reported among HIV-positive people. The largest number of new TB cases occurred in the Southeast Asian region with 44% of new cases, followed by the African region with 25% of new cases and Western Pacific with 18%. Eight countries accounted for two-thirds of the new TB cases including India (26%), Indonesia (8.5%), China (8.4%), Philippines (6.0%), Pakistan (5.7%), Nigeria (4.4%), Bangladesh (3.6%), and South Africa (3.6) [3]. *M. tuberculosis* does not have any particular virulence factor, but it perseveres long-term in the human body without causing any significant damage and transmission, if not, the immune system of the host is compromised. It secrets effector proteins to complicate the immune system, thereby, stimulating its intracellular survival in granulomas throughout the latency period of infection [4]. *M. tuberculosis* can develop in different conditions such as pulmonary and extrapulmonary TB (pleural, lymphadenitis, skeletal, gastrointestinal, and ocular) [5]. Once *M. tuberculosis* entered into the lungs, alveolar macrophages engulf the organism, and it was captured by phagosomes. Finally, it was delivered to lysosomes and get degraded. However, in most of the instances, to persist in human alveolar macrophages, the organism inhibits the acidification and phagosomes maturation. Also, to escape from the immune system recognition and hypoxic condition, the organism remains in a “quiescent status” (in nongrowing state but metabolically active) in 90% of the diseased individuals [6]. The innate (antimycobacterial elements, IFN*γ* and TNF*α*) as well as adaptive immunity (T cells, Th17, CD4^+^, and CD8^+^) controls *M. tuberculosis* activity in the latent phase of the infection. Hence, it is known as the conditional pathogenic bacterium [7]. If the host immune system is compromised, the organism gets activated and initiate replication. This promotes the diseased macrophage necrosis and, thereby, discharges the intracellular bacteria, which infect novel cells and invade other tissues [8]. Recent reports suggest that TB is also related with many other human complications, namely, autoimmune diseases (sarcoidosis: *M. tuberculosis* activates toll-like receptors, thereby, promoting pulmonary sarcoidosis [9]; systemic lupus erythematosus: immunosuppressive therapy and several immune abnormalities cause reactivation and diffusion of TB [10]), metabolic syndromes (diabetes mellitus: promotes the proliferation of mycobacterium [11]; atherosclerosis: mycobacterium rushes the development of atherosclerosis [12]), and pulmonary complications (pneumonia: TB infection rises the risk of secondary bacterial infection in children [13]; chronic obstructive pulmonary disease: pulmonary TB may alter the lung architecture [14]; lung cancer: TB is one of the risk factors for the development of lung cancer [15, 16]).

Treatment options available for drug-susceptible TB involve first-line drugs (isoniazid, rifampicin, ethambutol, and pyrazinamide) for six months. The indiscriminate use of the antibiotics leads to the development of resistance. The WHO classifies the resistance as multidrug resistance (MDR), extensive drug resistance (XDR), and total drug resistance (TDR). The rise of resistance to the first-line drugs termed as multidrug resistance (MDR) and resistance to the fluoroquinolones, one of the second-line drugs (capreomycin, kanamycin, and amikacin) tends to the occurrence of XDR. Both resistance to the first and second-line drugs leads to the development of TDR [3]. The U.S Food and Drug Administration (FDA) has developed bedaquiline, a novel drug against MDR *M. tuberculosis*. Over the 40 years, bedaquiline have been in use to treat TB. Unfortunately, resistance to bedaquiline antibiotic has been reported in recent years [17, 18]. The extrinsic factors associated with antibiotic resistance are social elements of TB in inhabitants and the eminence of prevention and control services of TB. Gygli et al., in 2017, explained the mechanism of intrinsic drug resistance in *M. tuberculosis*, such as decreased cell wall permeation, secretion of drug inactivating enzymes, mutation in the cell wall efflux system, bacterial drug target modification, and overexpression of drug targets. Here, isoniazid prodrug is activated by catalase or peroxidase (gene: katG), and it functions through enoyl acyl carrier protein reductase (gene: inhA). Rifampicin acts against *M. tuberculosis* through binding to the RNA polymerase *β* subunit (gene: rpoB). Ethambutol inhibits arabinosyl transferase (gene: embB) involved in the biosynthesis of cell wall arabinogalactan. Pyrazinamide, a nicotinamide analog, requires pyrazinamidase or nicotinamidase (gene: pncA) to get converted into pyrazinoic acid, an active form. For second-line drugs, fluoroquinolones inhibit the topoisomerase II (DNA gyrase), an essential enzyme involved in the replication process (genes: gyrA and gyrB). The role of kanamycin and amikacin modifies the range of 16S rRNA (gene: rrs) and, thereby, inhibits the protein synthesis. The capreomycin is found to inhibit the translation process in mycobacterium. The target gene tlyA participates in the rRNA ribose specific 2′-O-methylation. Here, the development of resistance to these first-line and second-line drugs is due to the mutations in the drug-targeting genes [19–23]. Such situation necessitates the design and development of novel drug with high antitubercular activity. In the recent study, Volynets et al., in 2019, have experimentally proved the activity of benzaldehyde thiosemicarbazone against *M. tuberculosis*. Hence, in the present study, we have retrieved the synthesized benzaldehyde thiosemicarbazone derivatives from the literature and screened against one of the main target DNA gyrases to find a potential inhibitor for *M. tuberculosis* [24] through molecular modelling methods. Our study may give an idea to the researchers who are designing drug against *M. tuberculosis* in the molecular level.

## 2. Materials and Methods

### 2.1. Protein Structure Preparation

**Figure 1:**
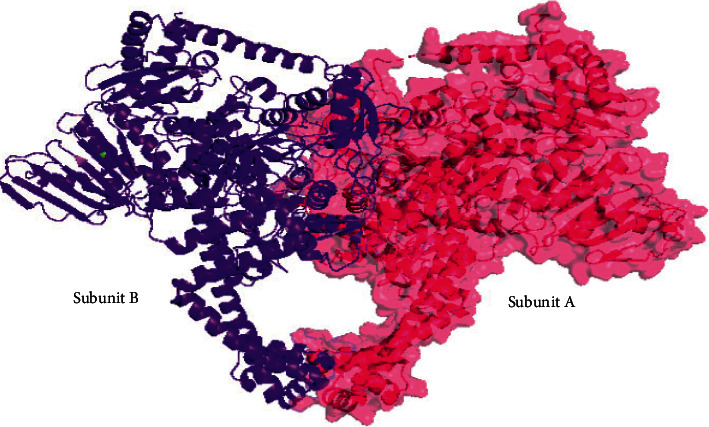
Structure of *M. tuberculosis* DNA gyrase.

The 3-dimensional (3D) structure of the target protein DNA gyrase was obtained from the protein data bank (PDB: 6GAU) [25, 26]. The PDB structure is a homodimer, which has two chains, chain A (DNA gyrase subunit B) and B (DNA gyrase subunit A) (Figure 1). For our virtual screening and molecular docking studies, we have utilized chain A. The sequence length of the subunit is 1179 amino acids. The 3D structure was crystalized by the X-ray diffraction method. The nonamino acid structures cocrystallized with DNA gyrase, phosphoaminophosphonic acid-adenylate ester, magnesium ions, and water molecules were detached from the structure.

### 2.2. Active Site Prediction

The active site of the DNA gyrase subunit B was predicted through the DoGSiteScorer [27]. Finding the low molecular weight ligand molecule by the target protein is the source for maintenance of the biological system. Here, active site is crucial for the function of an enzyme [28]. Usually, the active site prediction helps to ensure the protein function, druggability, and family classification. Such prediction is carried out by the DoGSite server, a structure-based prediction method, which functions on the basis of the difference of Gaussian (DoG) method. Most of the computational techniques available are geometry-based, energy-based, evolutionary-based, and combine method predictions. All of these approaches have disadvantages [29]. In contrast, DoGSite uses the pattern recognition method to predict the active sites. This locates the active site regions by sieving the grid of the protein using the DoG filter, and it finds the spherically designed structures called DoG cores in the grid. Finally, these detected DoG cores are gathered to form pockets, where the ligand molecules can fit firmly.

### 2.3. Ligand Preparation

**Figure 2:**
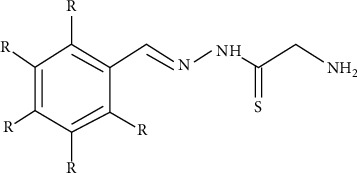
Structure of benzaldehyde thiosemicarbazone.

The synthesized 30 benzaldehyde thiosemicarbazone (Figure 2) derivatives were obtained from the literature (9). The structures were drawn with the help of ChemSketch software and optimized (10) (Table 1).

### 2.4. Virtual Screening

A total of 30 benzaldehyde thiosemicarbazone derivatives were screened against gyrB using the python prescription virtual screening tool (PyRx) [30], a structure-based virtual screening process, which usually screens compound libraries against protein targets. Using the OpenBabel tool combined with the PyRx server, the ligand molecules were added and subjected to energy minimization with the help of the universal force field (UFF) by the conjugate gradient algorithm. Both protein and ligand structures were converted into PDBQT format. The docking procedure was carried out through the AutoDock Vina of PyRx Tool. From the 30 compounds, best binding energy was selected and passed to the next procedure, docking studies to get final best compounds.

### 2.5. Molecular Docking Studies

Prediction of binding orientation of ligand with the protein is carried out through the molecular docking studies; in the current study, AutoDock version 4.2.6 was utilized [31]. It works on the basis of the Lamarckian genetic algorithm [32]. The target protein DNA gyrase B 3D structure was added with polar hydrogen atoms using AutoDock Tool (ADT). Both ligand and the protein structures were converted into PDBQT format. The grid was fixed with 80 × 80 x 80 size, which covers the active site of the protein. The desolvation map and grid map was created by the AutoGrid program. The algorithm was set with energy evaluation of 2500000, population of 150, 27000 generations, 0.02 mutation rate, and 0.8 crossover rate. Next, the AutoDock run was simulated, and the binding energy of protein-ligand affinity was checked to identify the best binding affinity. AutoDock produces empirical scoring functions; it is the combination of hydrogen bonding, van der Waals, electrostatic, hydrophobicity, entropy, and desolvation energies. The unit of binding energy is kcal/mol. The higher binding affinity between the complexes reflects the increased intermolecular forces. The lowest binding energy stabilizes the complex. The hydrophobic interactions were also explored using LigPlot+ v.2.2 analysis.

### 2.6. Absorption, Distribution, Metabolism, Excretion, and Toxicity Prediction

The pharmacokinetic properties such as absorption (A), distribution (D), metabolism (M), excretion (E), and toxicity (T) were predicted through the online server ADMET structure activity relationship (admetSAR). The database has structure and text search options, and also, the database collects, curates, and holds ADMET-related properties data from the available literature. The database includes 210,000 curated data for greater than 96,000 ligand molecules with 45 different ADMET-related properties, species, and proteins. The admetSAR interface is to search particular ligand profile, common name, and analog search. It has 5 quantitative regression models and 22 qualitative classifications with increased prediction accuracy. ADMET prediction is a distinctive interdisciplinary linking between the biologist, medicinal chemist, formulators, toxicologist, and regulators across India. The ADMET prediction of drug candidates has majorly reduced the drug failure in clinical trials [33].

## 3. Results and Discussion

**Figure 3:**
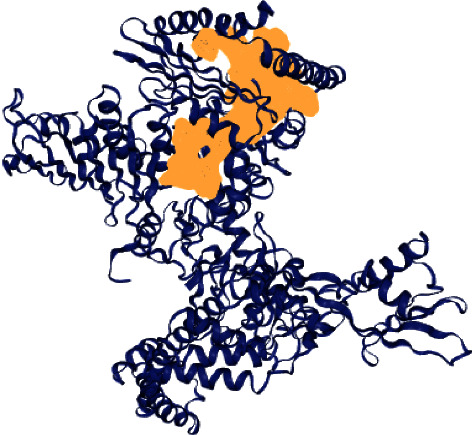
Binding pocket of DNA gyrase B.

TB, malaria, and acquired immune deficiency syndrome (AIDS) are the top most fatal infectious disease becoming a worldwide public health threat. The development of TB causes increased morbidity and mortality [34]. Different combinations of drugs have been given in treatment. There are several factors that have been reported for the failure of TB treatment, namely, delay in diagnosis, lack of effective drug administration, decreased accessibility of inexpensive, low toxic and effective drugs, prolong intake of drugs, lack of adherence to drug regimen, and rise of drug-resistant TB strains. Hence, to overcome the resistance mechanism, novel compounds were synthesized against the *M. tuberculosis*, and molecular modelling studies were carried out to find the potential drug candidate. The DoGSite server predicted the binding site pocket on the DNA gyrase B, which covered the area of 2834.65 Å^3^, surface 3418.38 Å^2^, and the drug score is 0.81. The drug score falls between 0 and 1, and the higher drug score reveals the high potential of the predicted pocket on the protein (Figure 3).

Virtual screening of 30 benzaldehyde thiosemicarbazone derivatives against DNA gyrase B reveals the high binding affinity of compound 13 with the binding energy of −8.5 kcal/mol (Table 1), followed by compounds 20 and 21 with the binding energies of −7.7 kcal/mol and −7.8 kcal/mol. Here, both compounds 20 and 21 have more or less similar binding energies. Other compounds, namely, 1, 5, 6, 7, 8, 9, 10, 11, 12, 13, 14, 15, 17, 19, 22, 24, 25, 26, 27, 28, 29, and 30 have their binding energies ranging from −6.0 to −6.9 kcal/mol. Also, the binding energies of compounds 2, 3, 4, 14, 16, 18, and 23 range from −5.6 to −5.9 kcal/mol. From Table 1, 10 best binding energy compounds were selected and passed through the molecular docking simulation studies using the AutoDock Tool.

**Figure 4:**
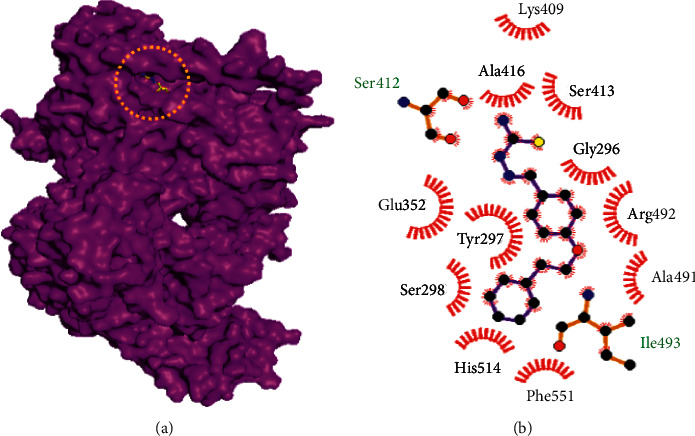
Binding mode of DNA gyrase B with the benzaldehyde thiosemicarbazone derivative compound 13. (a) Binding mode of compound 13 in the active site of DNA gyrase B. (b) Hydrophobic interactions of DNA gyrase B and compound 13.

**Figure 5:**
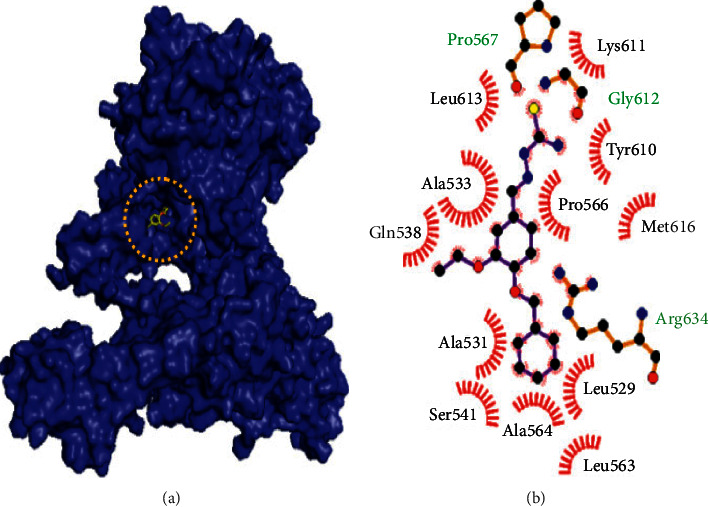
Binding mode of DNA gyrase B with the benzaldehyde thiosemicarbazone derivative compound 21. (a) Binding mode of compound 21 in the active site of DNA gyrase B. (b) Hydrophobic interactions of DNA gyrase B and compound 21.

**Figure 6:**
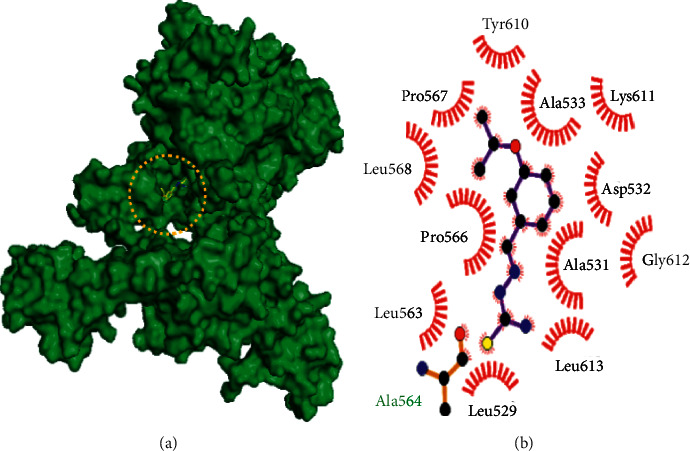
Binding mode of DNA gyrase B with the benzaldehyde thiosemicarbazone derivative compound 20. (a) Binding mode of compound 20 in the active site of DNA gyrase B. (b) Hydrophobic interactions of DNA gyrase B and compound 20.

Molecular docking studies of 10 best compounds against DNA gyrase B result in compound 13 as the best compound with the binding energy of −8.2 kcal/mol, which has formed four amino acid interactions with the bond distances of ILE493: 2.0 Å, SER413: 1.9 Å, LYS409: 2.8 Å, and SER12: 2.6 and 2.1 Å, and 11 hydrophobic interactions, namely, LYS409, SER413, ALA416, GLY296, TYR297, ARG492, ALA491, SER298, HIS514, GLU352, and PHE551 (Figure 4). Next, compound 21 is with −7.5 kcal/mol of binding energy, 3 hydrogen bond interactions such as GLY612 (2.1 Å), ARG634 (1.8 Å), and PRO567 (2.0 Å), and 12 hydrophobic interactions of MET616, LYS611, LEU613, PRO566, ALA533, TYR610, GLN538, SER541, ALA531, ALA564, LEU563, and LEU529 (Figure 5). Here, compounds 20 and 21 have only slight variations in their binding energies (Table 2). Compound 20 has the binding energy of −6.9 kcal/mol. The amino acids, namely, GLY612 (2.1 Å), ARG634 (1.8 Å), and PRO567 (2.0 Å) have been found in the hydrogen bond interaction. In hydrophobic interaction, the amino acids MET616, LYS611, LEU613, PRO566, ALA533, TYR610, GLN538, SER541, ALA531, ALA564, LEU563, and LEU529 have been found in the interaction (Figure 6). The higher the hydrophobic interaction, the lower the hydrogen bond interaction and vice versa. Our findings are similar to that of the experimental studies carried out by Volynets et al. [24]. The in vitro studies on benzaldehyde thiosemicarbazone derivatives against *M. tuberculosis* results in the minimal inhibitory concentration (MIC) of 0.68 *μ*M for compound 13 and 0.74 *μ*M for compound 20. In our findings, compound 21 is the second most high binding energy compound. Also, compounds 20 and 21 slightly vary in their binding energies.

Similar computational studies have been reported in the screening of antimicrobial compounds. Pertersen et al. [35] have used the virtual screening techniques to identify novel inhibitor for *M. tuberculosis* 3-dehydroquinate. Similarly, another study has employed pharmacophore-based virtual screening to identify the pyrazolo [1,5-*a*] pyrimidine derivatives against InhA of *M. tuberculosis* [36, 37].

Here, the first-line antimicrobial drugs rifampicin and isoniazid have lower energies −6.7 kcal/mol and −5.0 kcal/mol than the benzaldehyde thiosemicarbazone derivatives compounds 13, 20, and 21, respectively. The second-line drug levofloxacin is −7.0 kcal/mol.

From the virtual screening and molecular docking studies, it is confirmed that compound 13 could serve as a potential inhibitor for *M. tuberculosis*. Further preclinical studies have to be conducted to confirm the antimicrobial activity of the compound.

### 3.1. ADMET Prediction

ADMET prediction of benzaldehyde thiosemicarbazone derivatives using admetSAR server results in intestinal absorption capacity of the compounds 13, 21, and 20. These compounds also act as a nonsubstrate and noninhibitor for P-glycoprotein. Compound 13 serves as an inhibitor for renal organic cation transporter and other two compounds 20 and 21 as a noninhibitor. In the metabolism, for cytochrome P450 2C9, 2D6, and 3A4, the compounds 13, 21, and 20 possess nonsubstrate property and for cytochrome P450 1A2, 2C9, 2D6, 2C19, and 3A4, it results in noninhibitory activity. Toxicity prediction reveals the non-AMES toxicity and noncarcinogenic activity. Overall, the ADMET prediction further confirms the bioavailability of the compounds (Table 3).

## 4. Conclusion

The molecular modelling studies on 30 benzaldehyde thiosemicarbazone derivatives reveals the best binding energy of compound 13, compound 21, and compound 20 against DNA gyrase B of *M. tuberculosis.* To further confirm the activity of these compounds, preclinical studies have to be conducted.

## Figures and Tables

**Table 1:** PyRx (AutoDock Vina) virtual screening results for benzaldehyde thiosemicarbazone derivatives against DNA gyrase subunit B.

S. no.	Derivatives	Structure	Binding affinity (kcal/mol)
1	Compound 13	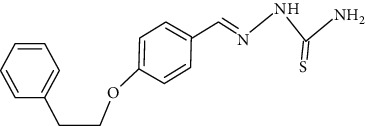	−8.5
2	Compound 21	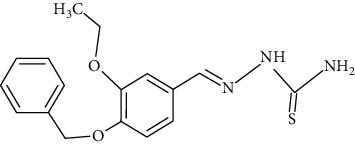	−7.8
3	Compound 20	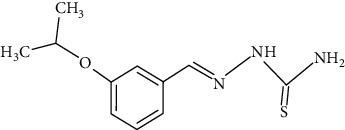	−7.7
4	Compound 5	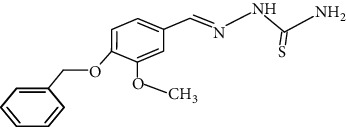	−6.9
5	Compound 7	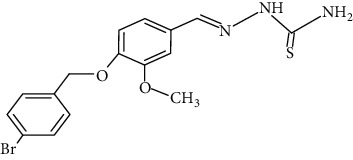	−6.9
6	Compound 1	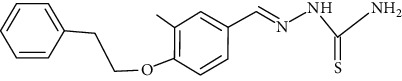	−6.7
7	Compound 26	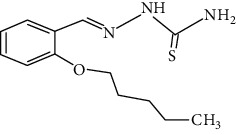	−6.7
8	Compound 6	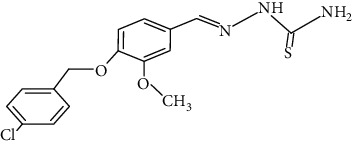	−6.7
9	Compound 28	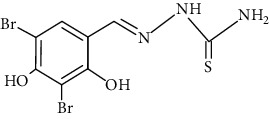	−6.6
10	Compound 15	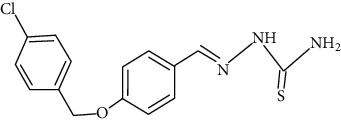	−6.6
11	Compound 9	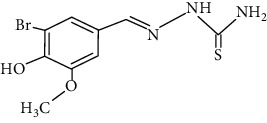	−6.5
12	Compound 27	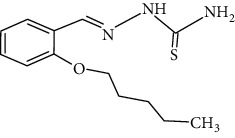	−6.5
13	Compound 10	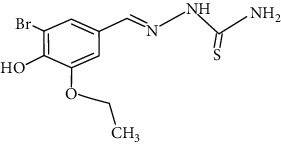	−6.4
14	Compound 25	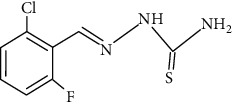	−6.3
15	Compound 19	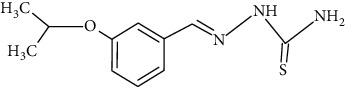	−6.2
16	Compound 29	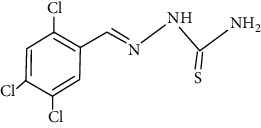	−6.2
17	Compound 24	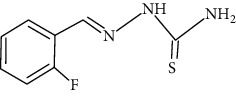	−6.2
18	Compound 30	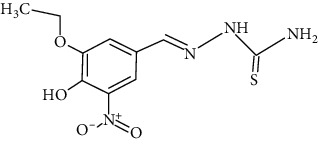	−6.1
19	Compound 8	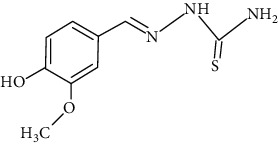	−6.1
20	Compound 11	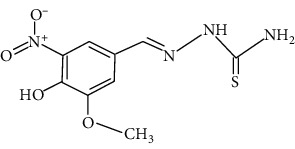	−6.1
21	Compound 12	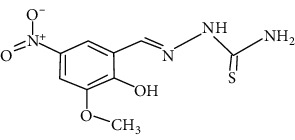	−6.1
22	Compound 17	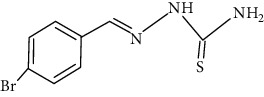	−6.1
23	Compound 22	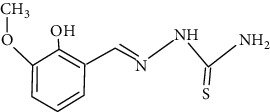	−6.0
24	Compound 4	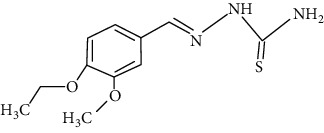	−5.9
25	Compound 14	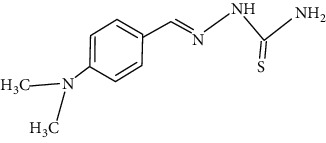	−5.9
26	Compound 23	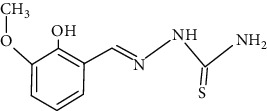	−5.9
27	Compound 2	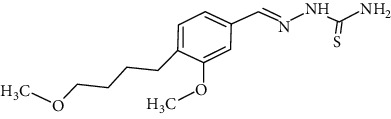	−5.8
28	Compound 3	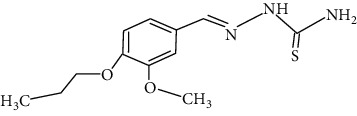	−5.7
29	Compound 18	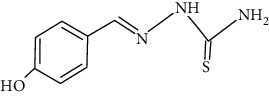	−5.7
30	Compound 16	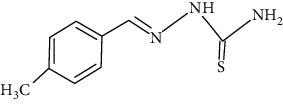	−5.6

**Table 2:** AutoDock results for benzaldehyde thiosemicarbazone derivatives against DNA gyrase subunit B.

S. no:	Compounds	Binding energy (kcal/mol)	Amino acid interactions and distances (Å)	Hydrophobic interactions
1	Compound 13	−8.2	ILE493 (2.0 Å), SER413 (1.9 Å), LYS409 (2.8 Å), and SER12 (2.6 and 2.1 Å)	LYS409, SER413, ALA416, GLY296, TYR297, ARG492, ALA491, SER298, HIS514, GLU352, and PHE551
2	Compound 21	−7.5	GLY612 (2.1 Å), ARG634 (1.8 Å), and PRO567 (2.0 Å)	MET616, LYS611, LEU613, PRO566, ALA533, TYR610, GLN538, SER541, ALA531, ALA564, LEU563, and LEU529
3	Compound 20	−6.9	ALA564 (1.9 Å) and ALA531 (2.0 Å)	TYR610, LEU613, ALA533, GLY612, PRO567, LEU568, PRO566, ASP532, LYS611, LEU529, LEU563, and ALA531
4	Levofloxacin	−7.0	ASN309 (2.5 Å) and ARG40 (2.0 Å)	LEU200, THR307, PHE304, GLU196, MET197, ASP639, TRP47, ASP640, ARG193, ASN309, ILE308, and HIS44
5	Rifampicin	−6.7	THR250 (2.3 Å), ASP259 (2.1 Å and 2.2 Å), and LYS262 (2.0 Å)	VAL301, ARG550, ASP348, HIS514, GLU317, HIS311, GLU312, PRO554, ASN558, and GLU557
6	Isoniazid	−5.0	SER1027 (2.7 Å), ALA531 (2.0 Å), LEU529 (2.1 Å), and ALA564 (2.2 Å)	ALA564, MET530, ARG634, LEU563, SER541, GLY537, GLU1023, and SER1027

**Table 3:** ADMET prediction for lead compounds.

S. no.	Models	Compound 13	Compound 21	Compound 20
Absorption				
1	Blood-brain barrier	BBB+	BBB+	BBB+
2	Human intestinal absorption	HIA+	HIA+	HIA+
3	Caco-2 permeability	Caco-2	Caco-2	Caco-2
4	P-Glycoprotein substrate	Nonsubstrate	Nonsubstrate	Nonsubstrate
5	P-Glycoprotein inhibitor	Noninhibitor	Noninhibitor	Noninhibitor
6	Renal organic cation transporter	Inhibitor	Noninhibitor	Noninhibitor

Metabolism				
7	CYP450 2C9 substrate	Nonsubstrate	Nonsubstrate	Nonsubstrate
8	CYP450 2D6 substrate	Nonsubstrate	Nonsubstrate	Nonsubstrate
9	CYP450 3A4 substrate	Nonsubstrate	Nonsubstrate	Nonsubstrate
10	CYP450 1A2 inhibitor	Inhibitor	Inhibitor	Inhibitor
11	CYP450 2C9 inhibitor	Inhibitor	Inhibitor	Inhibitor
12	CYP450 2D6 inhibitor	Noninhibitor	Noninhibitor	Noninhibitor
13	CYP450 2C19 inhibitor	Noninhibitor	Inhibitor	Inhibitor
14	CYP450 3A4 inhibitor	Noninhibitor	Noninhibitor	Noninhibitor

Toxicity				
15	AMES toxicity	Non-AMES toxic	Non-AMES toxic	Non-AMES toxic
16	Carcinogens	Noncarcinogens	Noncarcinogens	Noncarcinogens

## Data Availability

The data used to support the findings of this study are available from the corresponding author upon request.
